# Patient knowledge of HIV and its treatment in South Africa

**DOI:** 10.4102/phcfm.v6i1.518

**Published:** 2014-05-15

**Authors:** Lauren M. Terblanche, Ethelwynn L. Stellenberg

**Affiliations:** 1 Division of Nursing, Department of Interdisciplinary Health Sciences, Faculty of Medicine and Health Sciences, Stellenbosch University, South Africa

## Abstract

**Background:**

Patients on antiretroviral therapy (ART) need to achieve a 90% adherence rate to ART in order to prevent disease progression and drug resistance. The patients’ knowledge of ART and HIV is thus crucial to ensuring good adherence, decreased risk for drug resistance and cost-effective treatment for these patients.

**Aim:**

To determine the knowledge of infected patients with regard to HIV and the ART they were receiving.

**Setting:**

The study was conducted at a comprehensive community health centre in a developing low socio-economic community near Cape Town, South Africa.

**Methods:**

A quantitative descriptive correlative research design was applied. A sample consisting of 200 (8.5%) respondents was selected from a population of 2349. A multiple-choice questionnaire, comprising 29 questions, including 14 critical knowledge testing questions, was used in individual interviews conducted by either the researcher or fieldworker who assessed the respondents’ knowledge regarding various key aspects of HIV and ART.

**Results:**

Misconceptions regarding HIV and ART were revealed and scores for the 14 critical knowledge testing questions in the questionnaire revealed that 0% of the respondents had good knowledge, 20% had average knowledge and 80% had poor knowledge.

**Conclusion:**

The respondents on ART in this particular community health centre had poor knowledge of HIV and ART. This may contribute to poor adherence rates, increased drug resistance, disease progression and increased costs for the government with regard to treating such patients. Increased attention needs to be given to patient education.

## Abstrait

**Le savoir est essentiel: La Connaissance du VIH et de son traitement par les patients sous traitement antirétroviral.**

**Contexte:**

Les patients sous traitement antirétroviral (ART) ont besoin d'atteindre un taux d'observance de 90% à la thérapie antirétrovirale pour prévenir la progression de la maladie et la résistance aux médicaments. Par conséquent, la connaissance de l'ART et du VIH par les patients est essentielle pour assurer une bonne adhérence, réduire le risque de résistance aux médicaments et assurer un bon rapport coût-efficacité du traitement de ces patients.

**Objectif:**

Déterminer les connaissances des patients infectés sur le VIH et l'ART administré.

**Cadre:**

Cette étude a été menée dans un centre de santé communautaire polyvalent dans une communauté au statut socioéconomique faible près du Cap, en Afrique du Sud.

**Méthodes:**

Un modèle d’étude quantitative descriptive et corrélative a été utilisé. Un échantillon constitué de 200 (8.5%) répondants a été sélectionné parmi une population de 2349 individus. Un questionnaire à choix multiples composé de 29 questions, dont 14 questions testant les connaissances essentielles, a été administré au cours d'entretiens individuels menés par le chercheur ou l'agent de terrain chargé d’évaluer les connaissances des répondants sur divers aspects clés du VIH et de l'ART.

**Résultats:**

Des idées fausses concernant le VIH et l'ART ont été révélées et les scores pour les 14 questions du questionnaire testant les connaissances essentielles ont révélé que 0% des personnes interrogées disposaient de bonnes connaissances, 20% de connaissances moyennes et 80% de mauvaises connaissances.

**Conclusion:**

Les personnes sous antirétroviraux interrogées dans ce centre de santé communautaire particulier disposaient de mauvaises connaissances sur le VIH et l'ART. Cela peut contribuer à de faibles taux d'observance, à une augmentation de la résistance aux médicaments, à la progression de la maladie et à l'augmentation des coûts liés au traitement de ces patients pour le gouvernement. Une attention accrue doit être accordée à l’éducation des patients.

## Introduction

Despite the call for better coordination and monitoring in South Africa, the number of people living with HIV has shown a constant increase every year since the first diagnosis in the 1980s,^1^ with more and more people being affected by this debilitating disease. Studies have shown that the number of patients receiving antiretroviral therapy (ART) in South Africa has increased from less than 50 000 in 2004 to 1.79 million in 2011.^2^

Various interventions have been introduced with regard to reducing the vulnerability to HIV infection and the impact of acquired immune deficiency syndrome (AIDS) in order to increase awareness and knowledge of the disease, including:

- prevention of the sexual transmission of HIV through distribution of free condoms
- an increase in coverage for voluntary counselling and testing promotion of regular HIV testing
- the placement of non-profit organisation (NPO)-funded counsellors within community health centres (CHCs) to provide education and information in adherence classes as well as continuous HIV counselling and support to patients^1^.

However, research shows that South Africa's main contributing factors in the spread of this disease are rooted in poverty, underdevelopment, the low status of women and gender-based violence in the communities.^1^

The standard treatment of choice for people living with HIV is ART which functions by suppressing the replication of HIV.^3^ It has been confirmed (Mouton J. 2013, personal communication, January 16) that it currently costs the South African government approximately R124.00 per month to treat a patient with first-line ART and, once they move to second-line ART, approximately R547.00 per month. As there are currently only two lines of drug treatment in South Africa, it is important that patients adhere to their treatment, bearing in mind that patients who fail second-line therapy have few treatment options left to them.^3^ Adherence to ART is thus important with regard to containing the disease because incomplete adherence may lead to poor treatment outcomes and an increased risk for HIV-related mortality.^4^ Adherence of more than 90% needs to be achieved in order for ART to be effective.^5^ If ART is not adhered to correctly, HIV is not controlled optimally within the body – the viral load in the blood increases, thus aiding disease progression which poses a higher risk for opportunistic infections such as tuberculosis (TB).^6^ Poor adherence could also lead to ART resistance.^6^

Adherence rates are not always optimal, as was identified in a study conducted in South Africa^7^ which found that only 62.5% of patients had an adherence rate of more than 90% and, therefore, 37.5% of the respondents were at risk of developing resistance because of unacceptable adherence levels.^8^

It is believed that knowledge of the disease has an influence on adherence levels. Various studies have been focused on improving adherence through changing behaviour patterns and other interventions,^7^ such as patient education; however, there are few studies available that report on patients’ knowledge about various factors relating to HIV.^4^ Some studies conducted in specialised HIV clinics have shown that patients demonstrated good knowledge of HIV and the transmission thereof,^9^ but it has also been identified that adherence rates to ART are influenced strongly by misconceptions regarding HIV.^10^

Some misconceptions identified in studies conducted in Botswana were that out of 400 respondents, both male and female, 40.3% did not believe that they could be re-infected with HIV through sexual intercourse or any other means.^10^ Another misconception was that some people (*n* = 122; 30.5%) believed that HIV could be cured.^10^ In the same study it was indicated that 9.3% of the respondents believed that HIV did not even really exist.^10^

Another misconception revealed in a study conducted in KwaZulu-Natal, South Africa, was that showering after sex can be used as a preventative measure.^11^

It is believed that, with improved insight and knowledge about the disease and the management thereof, an improvement in adherence may result as patients with inadequate knowledge of HIV and its transmission would be more likely to report not taking ART.^12^

Orisakwe, Ross and Ocholla^11^ state that adherence classes provided by healthcare workers are vital for adherence to ART as they provide patients with information about the disease, correct doses of medication and possible side effects. It is believed that knowledge of ART and HIV will empower the HIV-positive individual. This is substantiated by the Health Belief Model which suggests that knowledge, attitudes and perceptions of risk affect the behaviour of individuals.^13^ HIV health education policies have been put in place by the South African government in order for healthcare workers and HIV counsellors to continue with HIV health education throughout the treatment of their patients.^1^ It is believed that the individual will consequently be motivated to be part of the collaborative process because initiation of ART is a clinical decision which should always be made jointly between the informed patient and the healthcare provider.^14^

Adequate patient education is specifically required when treating a patient infected with HIV as it comprises many facets that need constant attention. Patients need to be informed in full about their disease in order to be able to make informed decisions and to adhere effectively to treatment which would allow them to contain the disease. Patients may adhere to education given to them regarding prevention of HIV transmission through safe sex practices, taking medication as prescribed and accepting responsibility for their own health. This may, ultimately, contribute to an improvement in the quality of life of patients with HIV, as well as contributing positively to the strategic plan that the South African government has made in order to tackle the HIV problem in the country. For this reason, determining the knowledge that patients have regarding HIV may assist in determining whether or not the current patient education model being implemented in CHCs is successful and, if not, which measures can be put in place in order to improve the current model or implementation thereof.

The principle investigator (PI) identified during her clinical practice that the high burden of HIV in South Africa, combined with overcrowded CHCs, aggravated the daily management of patients. The high influx of patients into clinics every day increases time constraints and minimises consultation time, thereby diminishing the opportunity for effective patient education regarding HIV.^15^

With this in mind, this study set out to determine what knowledge infected patients receiving ART had with regard to HIV and the treatment they were receiving at the particular CHC, as well as to determine which demographic factors, if any, influenced this knowledge.

## Research methods and design

### Study design

This study followed a quantitative descriptive correlative research design.

### Setting

The study was conducted at a comprehensive CHC with a newly-built ART clinic in a developing low socio-economic community near Cape Town, South Africa with a population of 66 667.^16^ The ART clinic was equipped with doctors, nurses, HIV counsellors and other allied staff. The CHC managed an average number of 1200 patients daily presenting with a variety of medical conditions. This setting was chosen as it ensured a homogenous group of patients from the same socio-economic level.

### Study population and sampling strategy

The specific criteria for the target population for this study included all ART recipients who were above the age of 18, who had been on ART for at least three months and who were attending the particular CHC. The total number of patients who met the specific criteria for this study at the time was 2349.

As guided by a statistician, a convenience sample of 200 (8.5%) respondents was selected. It was decided that the first 200 respondents who voluntarily gave written consent to participate in the study and who complied with the specific criteria would be included in the sample.

Convenience sampling was best suited for this study because of the profile of the patients attending this CHC. Patients received booked appointments; however, various factors influenced the regular attendance of patients to the clinics (such as the availability of transport, unemployment and finances) and it could therefore not be relied upon that patients would attend their follow-up appointments.

### Data collection

A structured and pre-tested multiple-choice questionnaire which was designed by the PI was used for collecting the data. The questionnaire comprised two sections: Section A covered demographic data such as age and gender and also included the length of time living with HIV and the length of time on ART. Section B comprised 26 multiple-choice questions and three closed-ended questions which tested the knowledge of key aspects of HIV and ART such as modes of transmission, the duration of the window period as well as whether or not ART can cure HIV.

Twenty questions tested knowledge of HIV and ART, 14 of which were designated ‘critical questions’. The critical questions were identified in consultation with various HIV specialists as being the basic questions that all ART recipients should know and be able to answer.

The questionnaire was designed by the PI after an extensive literature review had been conducted on various aspects of the subject. The content validity was validated by the study supervisor, an expert in the field of research methodology and nursing, as well as by three additional experts in the fields of nursing, HIV and research. The questionnaire was then also reviewed by a statistician to ensure the suitability of the questionnaire for data analysis. A pilot study was conducted on 20 respondents in order to test the validity and reliability of the study. The questionnaire and methodology proved to be reliable and valid throughout both the pilot study and the main study.

The data was collected at the CHC over a period of three weeks in August 2011 by means of individual interviews with each of the respondents.

The fieldworker, a known educated Xhosa member of the community and a volunteer at the CHC, was trained. This included sitting in during five interviews conducted by the PI, which was done with the consent of the respondents. The fieldworker then conducted two individual interviews in the presence of the researcher before she was allowed to conduct interviews on her own. The fieldworker was fluent in both Xhosa and English.

Respondents were selected according to their availability and the willingness of each respondent to present for ART follow-up on a particular day; namely, those who complied with the specific criteria set for the study. Participation was completely voluntary and no incentive was given for taking part in the study.

Each respondent was provided with an information leaflet as well as a written informed consent document. Written informed consent was then obtained from each of the respondents, in their preferred language (English, Afrikaans or Xhosa) and, thereafter, the PI or trained fieldworker conducted an individual interview with each respondent, finally completing 200 interviews. It was stressed that participation was entirely voluntary and that, should the respondents agree to take part, they were free to withdraw at any time without having to give an explanation. Only two respondents excluded themselves because of time constraints.

The questionnaire was presented and completed by the PI or trained fieldworker during individual, structured interviews by reading questions in a language (English or Afrikaans) which the respondent understood, as well as providing all possible answers from the questionnaire from which the respondent could select answers. Interviewer bias was prevented by allowing the PI or trained fieldworker only to read a question and read out the set of answers slowly as stated in the questionnaire. The respondent was then given an opportunity to select an answer (or answers) which was marked off on the questionnaire by the interviewer. To ensure confidentiality, all documentation was anonymous, with no identification. Once the interview was completed, the completed questionnaire was placed in a box, which was with the fieldworker or PI at all times during the interviews. The PI collected the completed questionnaires from the fieldworker on a daily basis.

The data gathered from the study were handled only by the PI and the fieldworker, then coded and stored in a secure location.

Given the nature of the questions in the questionnaire, potential psychological stress was anticipated and there was always a counsellor available should respondents have required some assistance. However, none of the respondents experienced any stress during the process.

### Data analysis

Data were captured on an Excel spreadsheet by the PI and the document was then analysed with the help of a statistician, using the computerised data analysis programme, STATISTICA Version Nine (StatSoft, Tulsa, OK, 2009). For descriptive purposes frequency tables, histograms, means and standard deviations were used. Knowledge testing questions were correlated with the demographic variables. For comparison of variables, this included cross tabulation using the Chi-square test, correlation analyses or ANOVA. A 95% confidence interval with a significance level of (*p* ≤0.05) was used to establish statistically-significant associations between variables as discussed.

The questionnaire consisted of a total of 29 questions of which 20 tested HIV and ART knowledge. In consultation with various professionals in the field of HIV, 14 questions in the questionnaire were identified as critical questions (Tables 2 and 3) and it was expected that respondents should be able to answer these basic but important questions correctly. The other questions, however, were seen as ‘good to know’.

These 14 questions were identified to be the basis for deciding whether or not the respondents in the study had good, average or poor knowledge of HIV and ART. Respondents were considered to have good knowledge if they had all 14 critical questions correct, average if they had 11 to 13 correct and poor if they had 10 or fewer correct.

### Ethical considerations

Respondents were not exposed to any physical risks and permission to conduct this study was obtained in writing from the Health Research Ethics Committee of Stellenbosch University on 8 April 2011 (ref. N11/02/056) and from the Provincial Regional Head for Primary Health Care Services on 8 August 2011 (ref. LSB/ELS/08/11). Verbal permission was obtained on 9 August 2011 from the head of the CHC where the data collection took place.

### Results

The data indicated that 72.5% (*n* = 145) of the respondents were female and 75% (*n* = 150) were Xhosa speaking. The mean age was 37.5 years and, although the study was conducted in a low socio-economic area, 196 (98%) were literate with 78.5% (*n* = 157) having a highest education level between grades 8 and 12. Table 1 contains the demographic data from the study.

**TABLE 1:** Demographic characteristics and education of the study population (*N* = 200).

Variables	%	*N*
**Gender**	
Male	27.5	55
Female	72.5	145
**Home language**	
Xhosa	75	150
English	3.5	7
Afrikaans	18	36
Zulu	2	4
Other	2	4
**Literacy level**	
Can read and write	98	196
**Highest education level**	
No Schooling	1	2
Grade 1–7	20.5	41
Grade 8–9	22.5	45
Grade 10–12	56	112
**Length of time living with HIV**	
< 1 year	7.5	15
1–3 years	30	60
3–5 years	25.5	51
> 5 years	36.5	73
Don't know	0.5	1
**Length of time on antiretroviral therapy**	
< 1 year	23.5	47
1–3 years	39	78
3–5 years	20	40
> 5 years	17.5	35

*Note*: Minimum age 37.5; Mean age 37.5 years; Maximum age 62 years.

Knowledge regarding the action of HIV compromising the immune system was only answered correctly by 57% (*n* = 114) of the respondents and many of the respondents thought HIV merely destroys your organs; however, the spread of HIV was relatively well understood by most of the respondents (Table 2).

**TABLE 2:** Assessment of knowledge of HIV.

Questions	%	*n*
**Action of HIV (cc)**	
Kills the immune system†	57	114
**HIV is spread by (cc)**	
Kissing	96.5	193
Hugging	99	198
Sexual intercourse†	99	198
Mosquitoes	90	180
Coughing	88.8	177
Blood transfusion†	100	200
Infected bodily fluids†	94	188
**The window period is (cc)**	
When HIV cannot be seen in the blood†	42.5	85
**Duration of the window period (cc)**	
3 months†	41	82
**A CD4 count is (cc)**	
Number of fighter cells in the blood†	71	142
**How often a CD4 count should be monitored (cc)**		
12 monthly†	11.5	23
**Importance of monitoring the CD4 count**	
To monitor if treatment is effective†	69.5	139
**A viral load is**	
Number of HIV cells in the blood	35	70
**If you are HIV positive your children will definitely be positive (cc)**	
No	93.5	187

*n*, Results expressed as number of respondents with the correct answer; %, Results expressed as percentage of respondents with the correct answer;

† All correct answers; cc, Critical questions.

What is of concern, however, is that 1% (*n* = 2) of the respondents indicated that HIV cannot be spread through sexual intercourse and 6% (*n* = 12) stated that HIV cannot be spread through HIV-infected body fluids. Some had the mistaken belief that HIV could be spread through coughing and mosquitoes – this was observed during analysis (Table 2), which corresponded with previous studies conducted.

The knowledge of the window period was poor, with only 42.5% (*n* = 85) of the respondents knowing what it was.

Patients became very confused with the difference between a CD4 count and a viral load, giving the answer for what a viral load is under the question of CD4 count and *vice versa*. Only 71% (*n* = 142) of the respondents knew what a CD4 count was and only 60% (*n* = 120) knew what a viral load was. The majority (*n* = 159; 80%) of the respondents knew when their last CD4 count was done; however, few (*n* = 23; 11.5%) indicated correctly that their CD4 count should be measured every 12 months.

Most of the respondents were aware that HIV could be transmitted intrauterinally and 93.5% (*n* = 187) also knew that they could still have HIV-negative children with the help of ART.

The open questions allowed themes to be identified as to the reasons why respondents started on ART. It is interesting to note that, as previously mentioned, only 71% (*n* = 142) knew what a CD4 count was, but that 80% (*n* = 160) said that they were placed on ART because of a low CD4 count.

Respondents (*n* = 197; 98.5%) knew that ART could not cure HIV and that treatment was likely to be lifelong; however, only 93.5% (*n* = 189) of the respondents understood the actual function of ART. A misconception was identified in that 1% (*n* = 2) of the respondents indicated that ART has the function of making unprotected sex safe (Table 3).

**TABLE 3:** Assessment of knowledge of antiretroviral therapy.

Questions	%	*n*
**Antiretroviral therapy can cure HIV (cc)**	
No†	98.5	197
**Action of antiretroviral therapy (cc)**	
Kills the virus	2	4
Reduces the viral load in the blood†	94.5	189
Makes it safe to have unsafe sex	1	2
Don't know	2.5	5
**Patient received education on antiretroviral side effects**	
Yes	61	122
No	39	78
**Antiretroviral therapy can cause dangerous side effects (cc)**	
Yes†	36.5	73
**Name two danger signs (cc)**	
Could name two	10.5	21
**Action to take if experience danger signs (cc)**	
Stop and go to the clinic immediately†	63	126
Continue medication	3	6
Skip a day and continue	1	2
Don't know	33	66
**What should be done if medication is forgotten (cc)**	
Drink immediately when you remember†	76.5	153
**Result from ceasing to take medication (cc)**	
Antiretroviral therapy becomes less effective, HIV will increase	94.5	189

*n*, Results expressed as number of respondents with the correct answer; %, Results expressed as percentage of respondents with the correct answer;

† All correct answers; cc, Critical questions.

Education on side effects seemed to be lacking as many of the respondents indicated that they had not had any education on the side effects of ART.

Only 10.5% (*n* = 21) could name two danger signs and, although not all respondents (*n* = 126; 63%) could name the danger signs, they knew that if something abnormal did occur, they should stop the medication and go to the clinic as soon as possible. However, 33% (*n* = 66) did not know what to do at all.

The majority (*n* = 153; 76.5%) of the respondents knew that if they forgot to take their medication that they should take it as soon as possible thereafter.

Many of respondents indicated that ART did not cure HIV and when asked the question, ‘what will happen if you stop taking your medication?’, 94.5% (*n* = 189) of the respondents indicated correctly that ‘the ART will become less effective and the HI-Virus will increase in your blood’ (Table 3).

What was interesting is that most (*n* = 167; 83.5%) of the respondents indicated that they had gained their HIV and ART knowledge from the HIV ‘sister’ i.e. the professional registered nurse or the doctor (*n* = 11; 5.5%) in the clinic and not from other sources such as the HIV counsellors.

#### Respondents’ scoring

**FIGURE 1:**
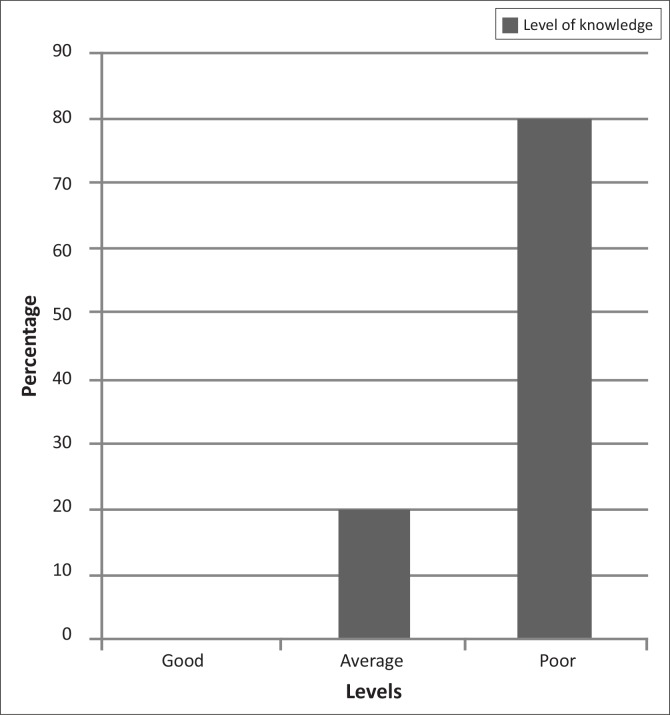
Analysis of the 14 critical questions, to determine level of knowledge.

Analysis of the data revealed that the average score of all 200 respondents for all 20 knowledge testing questions in the questionnaire, including the ‘good to know’ questions, was 63%. Analysis of the 14 critical questions revealed that none of the respondents had good knowledge, only 20% (*n* = 40) of the respondents had average knowledge and 80% (*n* = 160) of the respondents had poor knowledge (Figure 1).

## Discussion

The results show that the general knowledge of 80% (*n* = 160) of the study population was poor, contradicting results from previous studies.^10^

Furthermore, despite the fact that the study revealed that most ART recipients obtained their HIV and ART knowledge from the HIV nursing ‘sister’, the overall knowledge of HIV and ART amongst the recipients of ART was poor, contradicting the government's plan to decrease the incidence of HIV by promoting HIV education and counselling.

Basic terms and principles of HIV and ART were not understood by the respondents in this study and serious misconceptions regarding the disease were revealed, corresponding with results from previous studies.^2^ Men and women differed on occasion in the way they answered questions, however, demographic factors such as race and gender did not prove to have a significant influence on the overall knowledge of the respondents.

Despite the presence of HIV counsellors who function to give adherence counselling^1^ in the CHC under study, many (*n* = 78; 39%) respondents reported that they had not received any education on the side effects of ART and did not know that ART can potentially have dangerous side effects. This result makes one question whether or not patients were well informed and were part of the clinical decision to commence ART.^14^

A limitation identified was that the study was conducted in only one community and did not include any other communities. It was limited to the Cape Metropole area, therefore the results cannot be generalised. All respondents were able to speak and understand English, so language did not result in any bias.

Patient education is vital, as patients are enabled to take the information, carry the knowledge with them and make decisions based on what they know and have learnt. However, from this study it is clear that the HIV knowledge of ART patients at this particular CHC is poor.

Healthcare workers and HIV counsellors need to take up the role as a supportive-educative medium for the patient, by educating the patients and equipping them to make informed decisions based on what they know and understand. They, however, need to be informed, motivated and prepared to convey information and to make time for patient education using the resources available to them.

By identifying the deficit in patient knowledge, healthcare workers and HIV counsellors may decide which support modality is needed in order to educate and to promote knowledge and adherence. In addition, leaders in the community also need to be equipped with the knowledge and skills required to support and motivate members of the community with regard to HIV.

Education and health promotion empower people to take control of their own lives and their own health. This creates an environment which supports healthier lifestyles and informed decision making based on knowledge and understanding. Ultimately, this may contribute to an improvement in the quality of life of HIV-infected patients.

Based on the findings from this study, the following recommendations have been made:

1. Attention needs to be given to patient education in the clinical setting. Measures to improve patient education in this CHC need to be developed and implemented as soon as possible, especially since the research shows that patient knowledge is poor.
2. Education is essential with regard to facilitating patients to become mature individuals who are able to make informed decisions regarding their health and wellbeing.
3. Health workers and counsellors need to take note of who they are educating and what role each individual plays. Thus, they need to educate patients appropriately, as well as giving them any support necessary.
4. As the data revealed that most of the respondents’ knowledge was obtained from the HIV nursing ‘sister’, it is suggested that further research be conducted in order to implement an in-service training programme to further educate nurses on HIV and ART, followed by an evaluation process including monitoring of patient adherence statistics.
5. More research needs to be conducted in this clinical facility to determine the possible reasons for the poor knowledge of these particular patients.
6. The study revealed that there are gaps in research and it is thus suggested that:
  - further research should be done in other communities to determine if there are any similarities to the results found in this study and, should more poor knowledge trends be found in other sites, the current strategy for ART patient education should be revised
  - research also needs to be conducted to evaluate nursing staff's knowledge of HIV and ART
  - research should also be done to determine the factors that influence patient education in the primary healthcare setting.

## Conclusion

The results of this study revealed that of the respondents on ART at this CHC, 80% had poor knowledge of HIV and ART.

Despite the government's endeavours to increase awareness of HIV and ART in communities through patient education and counselling, as well as to implement patient education policies within the healthcare setting, it is disheartening to note that the majority of patients in this particular ART clinic had poor knowledge about HIV and ART, which indicates that health education is not a priority in this clinical setting. Higher priority needs to be placed on patient education and time needs to be set aside by all healthcare providers to educate patients at every visit and answer impending questions. Cost-effective treatment is reliant on the adherence of patients to their treatment, so by not educating patients about HIV and ART the cost for the government in treating these patients increases because of the occurrence of co-infections and drug resistance.

It is also imperative that health workers and counsellors dealing with HIV-infected patients, with support from the government, have up-to-date knowledge regarding all aspects of the disease and treatment so that they are able to convey the correct information to the patients they are treating.
